# Multifaceted Aspects of Dysfunctional Myelopoiesis in Cancer and Therapeutic Perspectives with Focus on HCC

**DOI:** 10.3390/biom14121496

**Published:** 2024-11-24

**Authors:** Andrea Arleo, Annapaola Montagner, Catia Giovannini, Fabrizia Suzzi, Fabio Piscaglia, Laura Gramantieri

**Affiliations:** 1 Department of Medical and Surgical Sciences, Bologna University, 40138 Bologna, Italy; annapaola.montagner@unibo.it (A.M.); catia.giovannini4@unibo.it (C.G.); fabrizia.suzzi3@unibo.it (F.S.); fabio.piscaglia@unibo.it (F.P.); 2 Division of Internal Medicine, Hepatobiliary and Immunoallergic Diseases, IRCCS Azienda Ospedaliero-Universitaria di Bologna, 40138 Bologna, Italy

**Keywords:** emergency myelopoiesis, dysfunctional myelopoiesis, aberrant myelopoiesis, cancer, immune system, inflammation

## Abstract

Myelopoiesis provides for the formation and continued renewal of cells belonging primarily to the innate immune system. It is a highly plastic process that secures the response to external and internal stimuli to face acute and changing needs. Infections and chronic diseases including cancer can modulate it by producing several factors, impacting proliferation and differentiation programs. While the lymphocytic compartment has attracted major attention due to the role of adaptive immunity in anticancer immune response, in recent years, research has found convincing evidence that confirms the importance of innate immunity and the key function played by emergency myelopoiesis. Due to cancer’s ability to manipulate myelopoiesis to its own advantage, the purpose of this review is to outline myelopoiesis processes within the tumor microenvironment and suggest possible therapeutic lines of research to restore the physiological functioning of the host’s immune system, with a special outlook on hepatocellular carcinoma (HCC).

## 1. Myelopoiesis Physiological Features

Myelopoiesis is a complex mechanism that provides for the formation and continued renewal of cells belonging primarily to the innate immune system. Physiologically, it takes place in the bone marrow, which is rich in hematopoietic stem cells (HSCs). Although HSCs were long considered merely standard stem cells, further studies have revealed their outstanding modulating influence on the immune system and a complex differentiation process [1]. In the bone marrow environment, HSCs generally spend much of their existence in a dormant (latent) condition, ending when HSCs divide for self-renewal or differentiate into a wide range of hematopoietic stem progenitor cells (HSPCs) to create effector cells. HSPCs are known to promote inflammatory processes and have been found to express high levels of CD34 and low levels of lineage markers. The factors and events responsible for initiating HSC differentiation are yet to be fully understood and require further investigation; however, it is widely agreed that the ultimate stage of this process is HSCs’ evolution into multipotent progenitors (MPPs), and some researchers have even argued that this might occur before cell division [2]. Recent studies on cell lineages have found that the von Willebrand factor (vWF) might force HSCs to produce exclusively platelets, while vWF-defective HSCs can differentiate into multiple lineages or fail to differentiate at all [3]. Although lineage commitment seems to occur randomly in traditional “tree-like” models, inherent cell biases have been found in single-cell profiling that steer the outcome of this commitment. Different populations of MMPs thus show a distinctive bias towards the production of a particular cell line [4,5,6], such as the megakaryocyte–erythroid-rich subpopulation (MPP2), the myeloid-abundant subset (MPP3), and a lymphoid cell line (MPP4). Sommerkamp and colleagues [7] have also described another similar subpopulation, MPP5, which they claim can generate MPP1 to MPP4 populations but cannot give rise to HSCs, calling for further studies to gain a wider comprehension of the process. Since these and many other different biases can be found in common MPPs, like myeloid ones, researchers have ascribed their multipotency to the low-resolution levels detected in surface markers [8,9].

### Myelopoiesis Differentiation Lineages

The HSPC differentiation chain leads to the rise of common myeloid precursors (CMPs), a type of oligopotent progenitors exclusively committed to the myeloid lineage and responsible for creating myeloid cell lines via specific myeloid precursors, namely megakaryocyte erythrocyte precursors (MEPs), monocyte dendritic cell precursors (MDPs), and granulocyte monocyte precursors (GMPs), which are ultimately responsible for the creation of mature immune cells [10]. MEPs give rise to mature cells that have lost their ability to perform mitosis, i.e., erythrocytes and megakaryocytes (MKs). The latter are characterized by a large, lobulated nucleus and constitute the source of platelets. Machlus and Italiano [11] have published an insightful review on the complex and fascinating process of platelet release, highlighting potential clinical applications for the creation of platelets from cultured MKs. MEPs seem to play a key role in high inflammation processes and studies on their involvement in cancer progression and immune escape are still underway, with promising results [12], as explained in the next sections. The second type of precursors, MDPs, are the source of cells that fight external menaces by means of phagocytosis. Since phagocytosis inevitably causes their death, there is an enduring need for a constant division of myeloid progenitors into differentiated cells that can replace these dead cells. This is important to balance the low proliferation activity shown by hematopoietic stem and progenitor cells and to prevent the loss of cells [13].

Here, we will focus on MDPs and GMPs, which play a relevant role in the tumor microenvironment. MDPs differentiate into two types of sub-precursors, namely common dendritic cell precursors (CDPs) and common monocyte precursors (CMoPs). CDPs are the source of dendritic cells, which play a critical role in antigen presentation to lymphocytes, whereas CMoPs eventually give rise to monocytes. While up to the CMoP differentiation stage, hematopoiesis processes take place in the bone marrow, monocytes migrate via the blood stream to other districts because of recruitment triggered by inflammatory processes. Here, they ultimately mature into macrophages, which are a resident tissue-related form of phagocytes and the first responders in case of infective agent attacks. However, downstream of CMoPs are also other populations of mature cells, including monocytic myeloid-derived suppressor cells (mMDSCs), which exercise a pivotal function in tumor immune escape processes, as further described in the next sections [14]. The third type of common myeloid precursors originated by HSPCs, GMPs, are responsible for creating three mature cell lines, namely neutrophils, basophils, and eosinophils. Besides their distinctive feature of a multilobed nucleus (hence their description as polymorphonuclear leukocytes), these cells are commonly referred to as granulocytes (as opposed to agranulocytes), because they are characterized by the presence of cytoplasmic granules containing lysosomal enzymes that digest invading pathogens. Granulocytes, especially neutrophils, participate in innate immunity, and tumors exploit this function to promote their growth and development, inasmuch as neutrophils can be recruited for immune escape activities and pre-metastatic niche formation [15,16]. Moreover, like monocytes originating mMDSCs, neutrophils can also give rise to granulocytic myeloid-derived suppressor cells (gMDSCs), which are known to be highly protumorigenic and capable of hampering adaptive, innate, and anticancer immunity [17,18].

When the organism faces stress or infections, it must produce a large number of immune cells in a short time. Such demands become even more intense in cases of chronic inflammation or solid cancer. Cells from the myeloid lineage, like macrophages and neutrophils, exhibit low proliferative activity due to their maturation process, which is strongly influenced by inflammatory signals. During active physiological hematopoiesis, pericytes, endothelial cells, and mesenchymal stem cells from the perivascular area of bone marrow regulate the differentiation of hematopoietic stem cells by means of cell-to-cell interactions and cytokines. To quickly counter prolonged inflammatory processes and solid cancers, hematopoietic stem cells (HSCs) can activate a process called emergency myelopoiesis, a mechanism that leads to the proliferation and maturation of myeloid cells, starting from the recognition of the threat by HSCs through Toll-like receptors.

There is no straightforward disambiguation in the existing literature between the terms *emergency*, *dysfunctional*, *aberrant*, and *abnormal* myelopoiesis, which are often used interchangeably or without a clear separation between the processes. Within the scope of this article, *emergency* myelopoiesis is to be understood as a physiological process resulting from any prolonged inflammation that leads to overactivated myelopoietic production. Only when this emergency process results in the production of immature immune cells that fail to fulfill their defensive function, we will use the term *dysfunctional* myelopoiesis. Finally, when dysfunctional myelopoiesis leads to the production of immune cells that are not only unable to fight cancer, but even promote their growth and proliferation, we will refer to it as *aberrant* myelopoiesis. It is worth pointing out that emergency myelopoiesis does not necessarily lead to dysfunctional myelopoiesis, as seen in cases of prolonged yet physiological inflammation. The same applies to the distinction between dysfunctional and aberrant myelopoiesis. Therefore, a clearer disambiguation of relevant terminology is all the more needed for accurately describing and investigating these phenomena.

## 2. From Cancer Immunoediting to Aberrant Myelopoiesis

**Figure 1:**
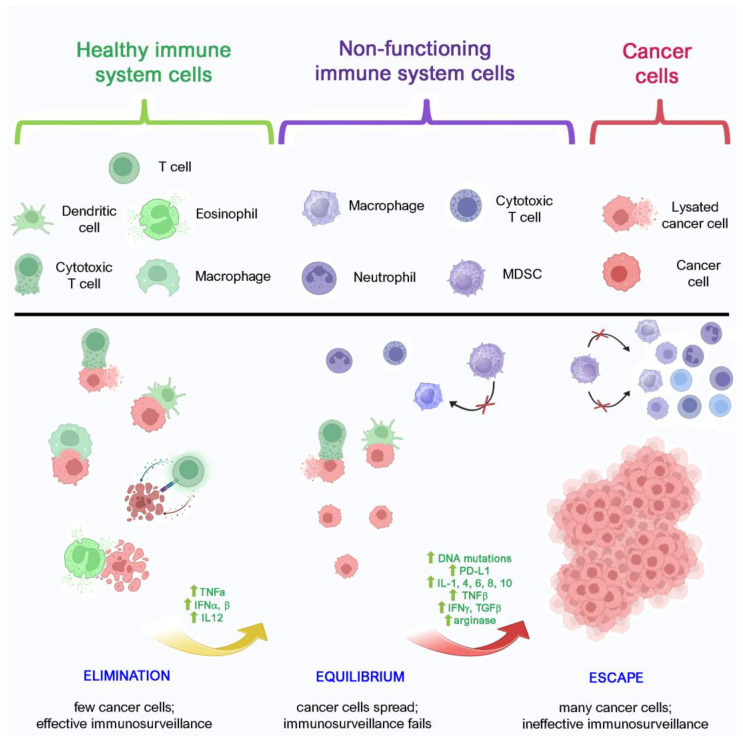
The immunoediting process, from the elimination phase to equilibrium and escape. During the elimination phase, the immune system is still able to eliminate aberrant cells, which start to overproduce TNFα, IFNα,β, IL12, and other cytokines. On the other hand, this makes aberrant cells stronger and harder to eradicate. A small amount of them survive, acquire DNA mutations, and start producing numerous cytokines which trigger PD-L1 upregulation. In turn, PD-L1 expressed by cancer cells binds the PD-1 receptor on lymphocytes, thus hampering their immune activity. As a consequence, immune cells lose their ability to recognize and effectively neutralize cancer cells. Eventually, immune cells become not only tolerant to cancer cells, but they are also recruited in the TME and produce cytokines that promote cancer progression, allowing cancer to spread and grow more and more aggressive. Functioning immune cells are represented in green, no longer functioning ones in purple, while cancer ones are depicted in magenta. MDSCs are depicted in purple since they actively contribute to inhibit immune activity, supporting cancer growth. Created with BioRender.com and then edited.

**Figure 2:**
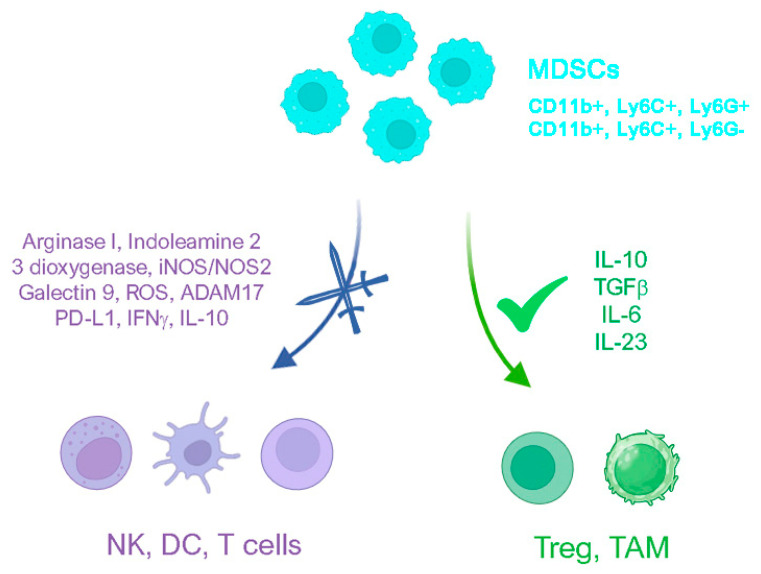
MDSCs are able to strongly influence immune system cells’ effects on an emerging tumor. MDSCs are able to produce a wide variety of cytokines that can fulfill different functions. On the one hand, they can hamper the functioning of immune system cells like natural killer cells, T lymphocytes, and dendritic cells; on the other hand, they can support the proliferation of T regulatory cells and tumor-associated macrophages, which can create a favorable microenvironment for cancer cells. MDSCs are depicted in cyan and non-functioning immune system cells are in purple, while functioning cells are in green. Created with BioRender.com and then edited.

Solid tumors are defined as the abnormal growth of cells within organs or solid tissues, originating from a small group of cells (or even one) which acquire a progressive series of mutations resulting in unstoppable mitosis. The carcinogenesis process implies a continuous fight for tumor survival and, to develop, cancer must profit as much as possible from those situations where it can be overlooked by the immune system. For this reason, it initiates a series of activities mainly targeted at bypassing immunosurveillance. This can be obtained by means of a three-step process called immunoediting, including elimination, equilibrium, and escape phases [19]. In the elimination phase, immunosurveillance is still efficient and yet able to kill nascent tumors. Both the innate and adaptive response collaborate to detect and eradicate malignant or transformed cells before clinical manifestation. As a matter of fact, at this stage, the presence of malignant cells can be extremely hard to detect at a clinical level, since they are quickly spotted and eliminated by cytotoxic immune cells like natural killer (NK) and CD8+ T lymphocytes. Nevertheless, some cells can exhibit low immunogenicity levels, so that the immune system is not able to recognize them. These escaped cells can survive but cannot grow in number, thus remaining in a balanced steady state either for short or long periods [20]. The process of genetic and epigenetic mutation accumulation is a sort of natural selection of cancer that enables the emergence of variants that are less immunogenic that can evade immunosurveillance [21]. Furthermore, it is possible to recognize some milestone events involving mutation and amplifications on oncogenes and tumor suppressors [22] and losses or gains of less or more extended portions of chromosomes [23] (Figure 1). During this phase, cancer can hijack immune activity to its own advantage to shape a favorable immunosuppressive tumor microenvironment (TME). Other two common events concurring with immune escape instauration are the blocking of the PD-1 receptor on immune system cells by means of its ligands, namely programmed death-ligand 1 or 2 (PD-L1 or PD-L2), expressed by cancer cells, and the recruitment of myeloid-derived suppressor cells (MDSCs) (Figure 2). PD-L1 is mainly expressed on cancer cells and on T and B lymphocytes. Both tumor intrinsic signals such as mTOR or MAPK signaling and microenvironmental factors like the activation of the IFN-γ cascade drive PD-L1 upregulation. The binding between PD-L1 and its receptor is a key event, since it is able to effectively turn off T-lymphocytes and it is physiologically used in order to cease the immune response, when it is no more needed. Tumors are able to take advantage of this mechanism and replicate it.

Moreover, the recruitment of immune system cells into the TME lays the conditions for the final stage, when cancer cells can largely escape immunosurveillance, and grow and spread without control. Since in both elimination and equilibrium, tumor cells tend to be completely obliterated, early diagnosis can only be performed during the escape phase.

The processes of immunoediting and the creation of an immunosuppressive TME deserve much attention and further studies, as a wide presence of immune cells are found in the TME as long as the cancer grows, with a number of clinical outcomes. For instance, high levels of macrophages in the stroma have been associated with a poor prognosis [24], while high levels of tumor-infiltrating T cells have been associated with a more favorable prognosis [25], especially in the case of cholangiocarcinoma, glioma, and breast cancer [26].

### 2.1. Aberrant Myelopoiesis and Cancer’s Hacking Activity

Cancer-related inflammation often begins with the secretion of proinflammatory cytokines. As a result, sustained cytokine signaling is needed to rapidly drive newly created immune cells to the tumor site and to maintain and strictly regulate their proliferation throughout the time required to eradicate the infection. This signaling involves molecules that favor: (i) tumor expansion, such as granulocyte colony-stimulating factor (G-CSF), granulocyte–macrophage colony-stimulating factor (GM-CSF), and macrophage colony-stimulating factor (M-CSF); (ii) tumor maturation, such as IL-1, IL-4, IL-6, IL-13, and TNF; and (iii) recruitment into the TME, such as IL-8, CCL-2, and CXCL12 [27,28,29]. Many of the cytokines present in the TME exhibit a proinflammatory action, like IFN-γ, while others, like IL-10, show anti-inflammatory effects [30]. Other molecules, like IL-27, are able to promote the repopulation of hematopoietic stem cells in the long term and their differentiation into myeloid progenitors. Similarly, IL-3 also exerts a strong action in maintaining hematopoietic stem cells.

When it comes to attracting lymphocytes to damaged tissues and CD8+ T cells to activated lymph nodes, CCL3 is essential. It carries out its activity by binding to its receptors, CCR1, CCR4, and CCR5. Together with other chemokines, CCL3 can elicit a strong inflammatory response. Moreover, it has been observed that in many cancer types, particularly chronic myelogenous leukemia and renal cell carcinoma, strong CCL3 production is correlated with the degeneration of the bone marrow microenvironment, facilitating tumor onset [31]. Furthermore, data show that CCL3 can also hinder hematopoietic progenitor proliferation, enhancing myelopoiesis in in vitro assays [32].

Interferons are powerful mediators capable of producing a wide range of effects. Type I interferons (α and β), primarily produced by monocytes and fibroblasts, generally induce apoptosis and have an antiproliferative effect on cells infected by viruses. However, these same molecules have shown the opposite effect on HSCs, inducing proliferation and exhaustion [33]. Meanwhile, type II IFN (γ), produced by activated NK and T cells, can transmit different signals depending on the other molecules present in the microenvironment, ranging from the inhibition of hematopoiesis [34] to the stimulation of HSC proliferation [35].

When oncogenes are activated, they can induce cellular senescence, which prevents tumor progression. However, these senescent cells can also produce proinflammatory cytokines such as IL-1, IL-6, and IL-8, which is known as the SASP, i.e., senescence-associated secretory phenotype [36]. It was observed that their production is strongly regulated by the NF-kB p65 subunit in oncogenic fibroblasts induced by H-Ras [37]. Often, a persistent inflammatory condition can be further reinforced by ancillary environmental factors such as cigarette smoking, hypoxia, obesity, inflammatory diseases, and microbial dysbiosis. Senescent tumor cells are also able to summon NK cells via CCL2. The cytokines regulating cellular senescence can also be influenced by tumor suppressors. For instance, it was observed that IL-1 receptor antagonists expressed by tumor cells can be inhibited by a mutant form of p-53 and, in doing so, the mutated p-53 can regulate the transition of cancer cells from a quiet state to an activated one [38]. This is a paradigmatic example of how cellular senescence works with a complex and sometimes antithetical outcome. SASP cells can be activated by the cGAS/STING sensor after spotting the presence of cytosolic DNA. This event leads to the production of type 1 IFN, boosting tumor suppression [39]. On the other hand, the continuous secretion of IL-1, IL-6, and IL-8, typical of a persistent inflammatory state, eventually results in effective immunosuppression and tumor escape [40]. Another interesting and concerning phenomenon caused by inflammation occurs when tumor cells are driven to undergo apoptosis and the debris they release triggers further cytokine production by macrophages [41]. This cytokine increase in the TME can fuel residual tumor cells, letting them thrive and spread. Similarly, systemic inflammation due to surgery can activate dormant cells, triggering tumor reactivation [42].

Another aspect affected by cancer’s hijacking activity involves the suppression of HSPCs, which starts already in the bone marrow, mediated by factors like TNF-α, TGF-β, SCF, arginase, and stanniocalcin, leading to emergency myelopoiesis. This condition, triggered by tumor-derived factors, is characterized by increased myelopoietic output, associated with the generation of myeloid populations endowed with tumor-promoting activities. This multistep process induced by tumors in turns fuels tumor growth, ultimately leading to the acquisition of a tumor-promoting phenotype by myeloid cells. Understanding these mechanisms might offer new therapeutic opportunities to counteract immune dysfunctions induced by aberrant myelopoiesis as well as overproduction of HSPCs in the spleen and other extramedullary sites in cancer patients [43,44]. Indeed, the disruption of hematopoietic processes physiologically occurring in the bone marrow can result in the production of HSCs in other sites, a phenomenon known as extramedullary hematopoiesis (EMH). EMH has been observed in the liver, lymph nodes, and spleen, but also, more rarely, in the pancreas, adrenal gland, skin, and pleura [45,46,47]. In so doing, hematopoiesis is hijacked by the TME so that it ends up creating and maintaining immunosuppressive cells that favor cancer growth, and a high number of HSPCs, which is known to correlate with an increase in the risk of metastatic progression [48].

#### 2.1.1. Erythropoiesis in the TME

Among other myelopoiesis processes, the interplay between erythropoiesis and cancer immunoediting activity is often overlooked. However, it is worth devoting more attention to the dynamics involved in this process, especially considering erythroid cells’ major involvement in angiogenesis [49] and their interaction with other myeloid cells, like macrophages, and inflammation-related molecules. Indeed, among other functions, macrophages support erythroblast proliferation and erythroblastic island (EBI) macrophages play a pivotal role in regulating the response of erythropoiesis to infections and inflammation [50], by releasing TNFα, IL-6, and IFN-γ [51,52]. Moreover, burst-forming unit–erythroids (BFU-Es) are known to interact with several growth factors and cytokines, such as the stem cell factor (SCF), IGF-1, glucocorticoids, and IL-3 [53]. In the case of a chronic inflammation, macrophages secrete proinflammatory cytokines that increase the production of hepcidin in the liver, a peptide hormone that binds to and inactivates ferroportin, preventing iron recycled by macrophages from erythrocyte, absorbed by enterocytes, and stored by hepatocytes to be delivered to plasma [54]. This exacerbates anemia and activates stress erythropoiesis, which in turn unbalances the differentiation of erythroids and remolds the TME to promote cancer progression and growth [55]. During cancer growth and progression, the spleen plays a key role as an immune cell reservoir including several cells with antitumoral activity, like T, B, NK cells, and macrophages. However, it has been observed that tumor-infiltrating erythroid progenitor cells are present in extremely higher numbers in both murine and human cancer patients [56] as compared to MDSCs and regulatory T (TReg) cells in hepatocellular carcinoma [57]. A study on mice by [58] showed that stress and hemodynamic changes induced by cancer result in an upregulation of erythropoietin, which triggers the hectic production of immature erythroids expressing genes that encode immune checkpoint molecules. Therefore, the use of anti-CD71 antibody to deplete EPCs resulted in a reduction in tumor-induced erythropoietin production and tumor growth. Indeed, besides interfering with EPC differentiation, the tumor-induced increase in erythropoietin production is also involved in the dynamics triggered by the vascular endothelial growth factor (VEGF), which is produced by cancer cells to enhance tumoral angiogenesis [59] and modulate the antitumoral immune response [60]. A pivotal role in the complex erythropoiesis process is played by hypoxia, which was recognized as a major trigger able to unleash the production of immature precursor, as further detailed regarding the aberrant production of MDSCs.

Erythropoiesis (and more generally hematopoiesis as a whole) is highly impaired by the tumor-induced dysregulation of the TGF-β signaling pathway, which not only hampers cell proliferation but also enhances the production of erythrocytes from HSCs and promotes mitophagy [61]. Moreover, the TGF-β signaling pathway is also involved in an IL-33–TGF-β niche triggered by tumor cells in the TME, affecting erythroid progenitor differentiation [62].

#### 2.1.2. Monocytopoiesis in the TME

Granulocyte-colony stimulating factor (G-CSF) is a cytokine able to promote the formation of granulocytes and stem cells in bone marrow via the activation of cEBPb [63] and STAT3 [64].

Aberrant dysfunctional monocytopoiesis is a hematopoietic process that plays an important role in the development of cancer, as monocytes influence both innate and adaptive immune responses and are deeply involved in cancer immunoescaping activity and in the provision of TME-infiltrating cells. Monocytes’ ability to migrate into extramedullary sites in response to inflammatory stimuli represents a powerful property that cancer can exploit to its own benefit, resulting in the recruitment of monocytes to differentiate in the TME [65] into tumor-associated macrophages (TAMs), tumor-associated dendritic cells (TADCs), and MDSCs.

##### Monocytes and TAMs

Monocytes and macrophages, along with other immune cells, express pattern recognition receptors (PRRs) that are able to recognize damage-associated molecular patterns (DAMPs) generated by cancer and trigger inflammatory response. The recognition of tumor DNA and RNA by STING, AIM2, and TL3, respectively, initiates the recruitment of monocytes to the tumor and metastatic niche, and an antitumoral immune response by T cells [66]. Although during early phases of antitumoral response, immune cells can still eliminate the tumor, the production of DAMPs and inflammation mediators in the TME leads myeloid progenitors to the induction of dysfunctional myelopoiesis. This results in the generation of immunosuppressive gMDSCs and mMDSCs, which are ultimately recruited in the TME and turned into TAMs [27].

TAMs can be found in the TME from the very early stages of disease and are able to promote a wide gamut of protumoral effects by cytokine production. They enable angiogenesis by means of VEGF, the epithelial–mesenchymal transition, immunosuppression by IL-10 and TGF-β, and tissue remodeling by means of metalloproteinases [67,68,69]. They can promote the reversion towards metastatic cells as well as the migration of cancer cells, thus stopping the production of macrophages and neutrophils in the bone marrow, which constitutes a pro-metastatic event that could be reverted upon macrophage elimination [70]. Due to their high ability to adapt to different TME conditions and stimuli, TAMs can highly differ from one another in different types of cancer and even within the same cancer type [71]. Moreover, the TME seems to steer TAM features regardless of their precursors [72], exploiting the ability of resident macrophages to overexpress genes involved in the remodeling of tissues, or monocyte-derived TAMs’ capability of overexpressing immunosuppression and antigen presentation genes [73]. TAMs can undergo phenotypic polarization resulting in two main phenotypes: the M1 phenotype, activated by IFN-γ and LPS, and the M2 phenotype, with subphenotypes M2a (activated by IL-4 and IL-13), M2b (activated by IL1β or LPS), and M2c (activated by IL-10, TGFβ, or glucocorticoids) [74]. M1 polarization is characterized by the expression of CD68, CD80, CD86, MHC-II, and iNOS. TAMs exhibiting this phenotype maintain inflammation so as to face acute symptomatology, thus performing tumoricidal activity linked to massive T cell recruitment. On the contrary, the M2 phenotype is characterized by the expression of Arg-1, VEGF, CD163, CD204, and CD206, thus providing growth factors for cancer and its endothelium, showing an evident ability to support immunosuppression. This is accompanied by the lack of cytotoxic activity, enabling a significant tumor growth-promoting action. M2 macrophages are also involved in inflammation resolutive processes, replacement and reparation of damaged tissues, and in the improvement of growth and motility of mesenchymal stem cells (MSCs) [75]. After the escape phase of immunoediting, tumors become able to recruit more and more immune cells. Gr1+ inflammatory monocytes (IMs) have been observed in pulmonary metastases, but not in the primary tumors, since they strongly enhance migration and proliferation. Since tumor and stromal cells bear CCL2 ligands, these monocytes are recruited due to the high number of expressed CCR2 receptors for CCL2 chemokine. Interestingly, blocking this receptor system results in stopping IM recruitment, evident metastasis reduction, and a better prognosis for breast cancer [76]. During long-lasting CCL2-induced IM recruitment, there is an evident switch of resulting macrophages from M1 to M2 phenotype population. One of the most studied agents that is able to influence dysfunctional monocytopoiesis and cancer progression is the retinoic acid-related orphan receptor (RORC1/RORg). This molecule plays a major role in MDSC and TAM differentiation, survival, and switching into their M2 phenotype [77]. Furthermore, RORC1/RORg is also able to activate monocytopoiesis in a threefold way: first, by hampering negative effectors like Socs3 and Bcl3; second, by enhancing positive modulators like C/EBPb; and eventually, by stimulating the production of PU.1 and IRF8, which are known for their ability to determine the commitment and differentiation of myeloid progenitors into fully differentiated cells from a monocytic/macrophagic lineage [78]. A study conducted by Strauss et al. [77] showed that the pharmacological or genetic blocking of RORC1 results in a dramatic reduction in MDSC growth, M2 macrophage phenotype switching, and decreased tumor development.

##### TADCs

Dendritic cells are the most effective cells in presenting antigens to naive T cells. Their presence in TME is correlated with a better prognosis in breast, lung, and head and neck cancers [79] and also a better T cell response [25]. It is of utmost importance to note that TADCs are strongly involved in the creation of a new, tertiary lymphoid structure (TLS) within the tumor, originating from either the barrier or the tumoral core after prolonged exposure to inflammatory cytokines. Undeniably, this is a powerful site for tumoral antigen presentation, especially due to the abundance of B lymphocytes in the TLS [80]. Like other cell populations trackable in TME, TADCs also show two different phenotypes. Type 1 cells are able to activate T cells, to present tumor antigens, to produce high levels of IL-12, and to enhance CD8+ T cell growth. On the other hand, Type 2 DCs are able to activate CD4+ T cells via the major histocompatibility complex II (MHC-II). The transition that brings from monocytes towards TADCs occurring in the TME determines the acquisition of new membrane markers like CD11c, CD80, CD86, and CD103 [81]. Notably, in different cancer types, TADCs can acquire protumoral or, conversely, antitumoral effects and the final outcome will be strongly influenced by the intrinsic characteristics and composition of single TMEs.

##### MDSCs

Myeloid-derived suppressor cells (MDSCs) are a diverse and multifaceted cell lineage during dysfunctional myelopoiesis involved in the suppression of antitumor responses [82] and their differentiation path is still not completely characterized, although a surface-marker-based classification distinguishes between monocytic, granulocytic, and immature MDSCs [83]. MDSCs produced in the bone marrow are equipped with CCR2 receptor and are then recruited into the TME by tumor cells expressing CCL2 ligands, with a possible involvement of the hypoxic conditions that characterize the TME [84,85]. Indeed, hypoxia has been found to play a critical role in myeloid differentiation within the TME. Hypoxia is a prominent condition in the TME that promotes the differentiation of M-MDSC into TAMs as a result of pSTAT3 downregulation [27]. The disruption of MDSC differentiation process is mediated by the hypoxia-inducible factor (HIF)-1α, whose absence makes MDSC exhibit markers typical of dendritic cells [86,87]. Moreover, hypoxia has been observed to enhance the expression of PD-L1 in tumor-infiltrating MDSCs, TAMs, and dendritic and tumor cells, thus enhancing tumor-suppressive activity [88].

As in the case of other lineages resulting from the emergency myelopoiesis (EM) process, MDSC activation and proliferation are promoted by several autocrine and paracrine chemokines, often secreted by tumors, including IL-6, IL-1b, macrophage- and granulocyte-colony stimulating factor (M-CSF, G-CSF), VEGF, and beta fibroblast growth factor (β-FGF) [89]. Out of the many functions these molecules serve, a shared feature is that they all co-occur with the activation of a powerful transcription factor of MDSCs known as signal transducer and activator of transcription 3 (STAT3) [90]. STAT3, along with the Janus kinase (JAK), is able to regulate the transcription of a great number of genes, including the ones acting on myeloid progenitor cells, such as cyclin D1, MYC, and survivin [91]. Moreover, the combined effects of all the aforementioned factors alter myeloid cells’ maturation, cause the promotion of epithelial–mesenchymal transition (EMT) and mesenchymal–endothelial transition (MET) [92], which enhance tumor angiogenesis and metastases, negatively impacting antitumor therapies and overall survival [93]. Moreover, this is accompanied by increased production of immature myeloid cells and MDSCs [94,95,96], which favors immune tolerance and cancer stemness, with critical implications for patient outcomes [97]. Indeed, MDSCs can anergize T and NK cells by regulating the overproliferation of amino acid catabolic enzymes (e.g., Arginase I, Indoleamine 2,3 dioxygenase, iNOS/NOS2), galectin 9, reactive oxygen species (ROS), ADAM17, and Treg cells [94]. This immune-suppressive process is the result of MDSCs’ restraining action on cystine, which is strongly involved in T cell activation. Indeed, under inflammatory conditions, macrophages increase the production of cystine and release cysteine, which is then sensed by T cells [98]. Therefore, immune evasion resulting from anergic T and NK cells could be established by means of amino acid depletion by MDSCs. Another mechanism MDSCs can implement to impair T cell response is the expression of high levels of PD-L1, resulting in the production of two inflammatory proteins, namely HIF1a and S100A9, and in the induction of aberrant myelopoiesis [99]. Furthermore, the activity of T and NK cells can also be affected by the underproduction of IL-10 and IFNγ, due to MDSCs’ expression of CD40 receptors and immune-suppressive TGFβ cytokines [100]. Due to the key role that MDSCs play in cancer-associated immune suppression and the subsequent negative response to treatments, MDSCs constitute a promising target for cancer therapies [101,102] to inhibit their action and restore the immune-suppressive microenvironment.

#### 2.1.3. Granulopoiesis in the TME

Granulopoiesis is a continuous process occurring in the bone marrow to restock migrated granulocytes like neutrophils, basophils, and eosinophils. Under physiological conditions, colony-stimulating factors (G-CSF, M-CSF, and GM-CSF) and transcription factors regulate the normal maturation of granulocytes, but the dysregulation resulting from tumor-secreted factors leads to the production of granulocytic (or polymorphonuclear) MDSCs (PMN-MDSCs) [103,104]. The overproduction of PMN-MDSCs is favored by a number of transcription factors, including IRF8, STAT3, STAT5, C/EBPβ, and β-catenin [105,106,107]. PMN-MDSCs are thus recruited into the TME to favor tumor growth and immune suppression in different types of cancer [108].

The role of granulocytes in cancer is garnering increasing attention, due to observations in both clinical and preclinical studies that patients with advanced and aggressive cancer exhibit an increased number of circulating neutrophils and tumor-infiltrating neutrophils (TANs), especially in the case of aggressive tumors [109]. This increased neutrophil-to-lymphocyte ratio (NLR) correlates with low survival rates and serves as a simple biomarker in a number of diverse cancers, including hepatocellular carcinoma [110,111]. As in the differentiation of TAMs into M1 and M2 phenotypes, Fridlender et al. [112] found that TANs also undergo tumor-mediated transformation in the TME into antitumoral N1 and protumoral N2 TANs. However, it has been recently suggested that these might be different maturation stages rather than phenotypes [113]. TAN plasticity has been shown to be regulated by IFN-1, promoting differentiation into N1 TANs. Conversely, N2 TAN polarization is induced by GCSF and IL-6 [114], as well as impaired IFN-1 [115]. This N2 TAN population is known to favor tumor progression, angiogenesis, and metastasis, due to TANs’ ability to secrete factors including oncostatin M, matrix metalloproteinases, and hepatocyte growth factor [116,117].

Out of all granulocytic cells, neutrophils are undoubtedly the most numerous type of circulating immune cells, constituting up to 70% of all peripheral circulating leukocytes, with over 50% of the bone marrow dedicated to their production [118]. For this reason, other granulocytes tend to be overlooked in research, as in the case of basophils, which constitute less than 1% of human leukocytes. Although they were long regarded as effectors merely involved in allergies, studies have found that they possess the ability to infiltrate inflammation sites [119,120] and produce several cytokines, including IL-3, IL-4, and IL-13 [121,122,123]. Human basophils have been found to produce a range of angiogenic factors, including ANGPT1, VEGF-A and VEGF-B, HGF, and CXCL8 [124,125,126,127]. Moreover, basophils can form extracellular DNA traps, which are known to promote tumor growth, metastasis, and the awakening of dormant cancer cells [128]. As in the case of neutrophil-associated NETs and TANs, they might constitute a potentially exploitable therapeutic target. Their involvement in cancer-mediated immune regulation is also made plausible by their ability to express PD-L1 in the TME [129]. Targeted by checkpoint blockade immunotherapies to trigger antitumor immunity, the expression of PD-L1 in immune cells within the TME, rather than on tumor cells, is critical for the effectiveness of these therapies [130,131]. However, studies on basophils are usually performed under normal conditions and further research is needed to unveil the characteristics of basophils in the TME and the role of PD-L1 in releasing basophils in immunotherapy.

**Figure 3:**
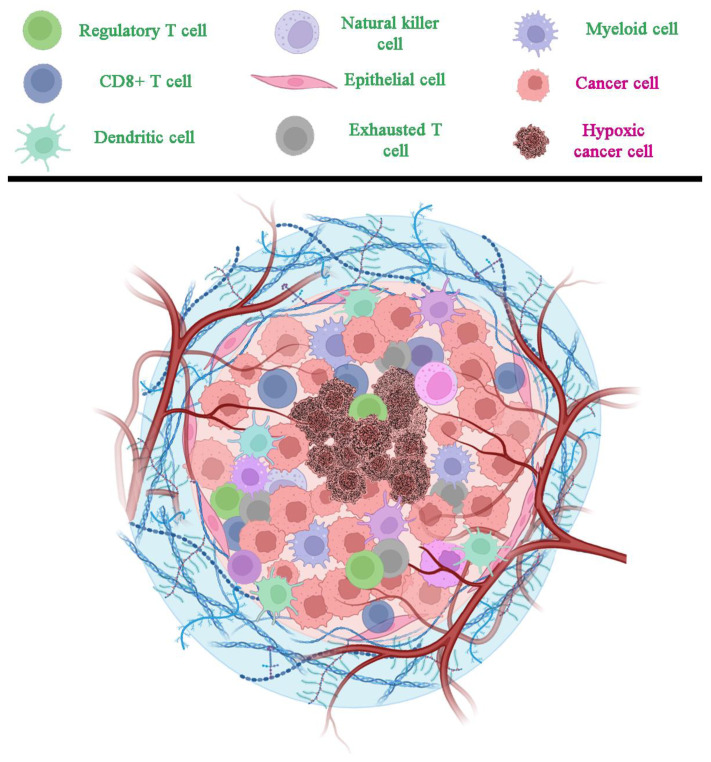
Cells shaping the TME. The inner part of a developing cancer is composed of dead or suffering cells due to hypoxia. They are surrounded by a mass of frantically replicating cells requiring a large number of quickly grown blood vessels that often exhibit a messy structure. Among these continuously growing cells, a large number of lymphocytes and other immune cells are recruited, producing a wide gamut of cytokines, signaling molecules, and growth factors (not shown) that are necessary for the evolving tumor. In order to highlight the presence of a senescent fraction in different cell lines, senescent cells are depicted in purple. Created with BioRender.com and then edited.

Like basophils, eosinophils also constitute less than 1% of all human leukocytes, but they have been observed in the TME of different human solid and liquid tumors, although the mechanisms by which they infiltrate tumor tissue have yet to be uncovered [132,133]. IL-5 and GM-CSF, also produced by cancer cells, have been observed to attract eosinophils and trigger their migration into the peripheral blood flow [134,135]. In vivo studies on IL-4 have shown its antitumor activity by recruiting eosinophils into the TME [136]. Several studies have claimed that eosinophils might exert antitumor activity in different tumors, including colon cancer [137], hepatocellular carcinoma [138], and melanoma [139]. Indeed, in the case of melanoma, eosinophils have been found to secrete chemokines like CXCL10, CCL5, and CCL9, which can recruit CD8+ T cells to the TME, and can promote M1 polarization of macrophages by secreting TNF-α and IFN-γ [140]. In HCC, a low baseline eosinophil count is associated with a poorer prognosis and its negative prognostic impact was also confirmed in the setting of systemic treatments [141]. Additionally, a study on a melanoma model observed an IL-33-induced decrease in cancer growth due to the invasion of CD8+ T cells and eosinophils in the TME and found that eosinophils are able to exert a direct cytotoxic action on tumor cells in vitro [139]. In light of these observations, some studies have investigated eosinophils in cancer immunotherapies, finding that stimulation with IL-2 resulted in increased levels of eosinophils and their cytotoxicity on cancer cells [142]. Recent therapies targeting immune checkpoints like PD-1, PD-L1, and CTLA4 have shown that anti-CTLA4 treatment resulted in an increase in eosinophils, which correlates with increased survival [143]. However, since eosinophils have been observed to express both PD-L1 and PD-L2 [144], further investigations are needed to unveil the interplay between eosinophils, PD-1/PD-L1, and the TME (Figure 3).

## 3. Emergency Myelopoiesis and Metabolic Syndrome

The term “metabolic syndrome” encompasses several inflammatory conditions such as obesity, hypertension, insulin resistance, and dyslipidemia [145] and often triggers inflammatory events, overactivating and dysregulating the immune system and eventually leading to immune deficiency. Although the interplay between metabolic signaling and subsequent immune response is still to be fully uncovered, studies on colorectal cancer have shown that the metabolic syndrome significantly affects lymphocyte phenotypes, their development, and activity. These effects correlate with an increased risk of cancer recurrence [146,147].

The alteration of HSCs and myeloid progenitors is a shared characteristic of cancer and metabolic disorders, serving as an effective biomarker for obesity-related conditions like diabetes and atherosclerosis [148,149]. Studies on the relationship between obesity and cancer have found that tumors can hinder oxidative phosphorylation and promote glycolysis [150], leading to enhanced expression of granulocyte and macrophage colony-stimulating factors, as well as NF-κB, which foster increased myelopoiesis and MDSC differentiation [151]. Moreover, obesity favors cancer progression by creating a strong immunosuppressive environment with enhanced production of MDSCs [152], as observed, for instance, in renal carcinoma [153]. Observations on diet-induced obese murine models of pancreatic ductal adenocarcinoma (PDAC) have revealed that obesity is associated with accelerated tumor growth and impaired chemotherapy effectiveness, due to the recruitment of tumor-associated neutrophils (TANs) by adipocyte-secreted IL1β, resulting from overactivation of pancreatic stellate cells [154].

As previously described, under physiological conditions, HSCs do not undergo massive division but their rapid and hectic proliferation can be triggered by inflammation, also in obese hosts [155]. Indeed, obesity significantly alters metabolism, leading to chronic inflammation. Studies have shown that the co-occurrence of chronic inflammation and insulin resistance correlates with a higher risk of cancer [156]. Metabolic alterations appear to affect patients’ response to chemotherapy in several ways. For example, insulin-like growth factor (IGF-1) is known to interact with NF-κB signaling [157], and high circulating levels of IGF-1 have been observed to promote an inflammatory TME, resistance to apoptosis [158,159], and chemoresistance [160]. Moreover, hyperglycemia, which is a common feature of type 2 diabetes mellitus and obesity, has been identified as a factor affecting the development and prognosis of various types of cancer [161] and conferring resistance to drug-induced apoptosis in both breast and prostate cancer [162,163]. In fact, hyperglycemia and hypercholesterolemia can affect HSPC proliferation, especially in patients with diabetes [164,165], by enhancing the massive production of neutrophils and monocytes [166]. Lipid and cholesterol metabolisms are significantly influenced by retinoic acid-related orphan receptor (RORC1/RORγ), which is associated with enhanced tumor-promoting aberrant myelopoiesis and increased proliferation of immature MDSCs, while its depletion triggers a strong T cell response and cancer regression [77]. However, some aspects of the interplay between metabolism and the TME remain controversial. For instance, a high body mass index (BMI) has been observed to impact the outcome of immunotherapy for advanced tumors, as BMI-induced high levels of fatty acids, glucose, insulin, and IGF-1 do promote cancer progression but can also foster immune response [167]. Similarly, MDSC expansion in patients with metabolic syndrome and subsequent dysregulation of myelopoiesis is a topic of ongoing debate. On one hand, MDSC proliferation in patients with obesity and metabolic syndrome is accompanied by dysfunctional and hyperactive myelopoiesis, potentially leading to a lethal cytokine storm and subsequent multi-organ system dysfunction [168]. On the other hand, studies on murine models have found that MDSCs can serve a protective function against inflammation and metabolic dysfunctions [169,170].

A thorough understanding of the interplay between human metabolism, cancer, and TME-induced effects on myelopoiesis and the immune system is critical for informing research on cancer immunotherapies. For instance, immune checkpoints like PD-1 can induce metabolic remodeling of myeloid precursors and enhance MDSC proliferation [171]. Moreover, metformin can be used as a complementary therapeutic agent in cancer treatment to reduce cancer incidence and mortality, mitigate the damaging effects of androgen derivatives, and increase the response to chemo- and radiotherapy [172], thus paving the way for further research on the metabolic effects of this and other recent drugs targeting metabolic dysfunction on myelopoiesis and cancer.

## 4. Perspective for Cancer Immunotherapies and the Case of Hepatocellular Carcinoma

Considering the major interplay between myelopoietic processes and cancer immunosuppressive activity, the need for targeting undifferentiated circulating immune cells such as MDSCs and HSPCs emerges as an issue to be addressed for cancer treatment. Several actions can be undertaken to try to limit the damages resulting from these tumor-infiltrating cells. Treatments can aim to (i) increase the general differentiation and level of commitment of MDSCs and HSPCs; (ii) lower the dedifferentiation degree of HSPC cells into MDSCs; (iii) hinder MSCs’ immune suppressive ability; (iv) manipulate and modify effector cells by equipping them with new engineered antigens as in CAR-T therapy; (v) enhance myeloid effectors’ antigen presentation activity in the tumor; and (vi) reactivate immune-checkpoints in order to rescue exhausted lymphocytes. Immunotherapy based on immune-checkpoint modulation is a powerful therapeutic approach that has emerged over the last fifteen years and has demonstrated strong results both in terms of overall survival rate and tumor reduction. Nevertheless, immunotherapy has shown to be particularly effective for certain types of tumors, for instance, bladder, lung, colorectal cancer, and melanoma [173].

HSCs were also studied in combination with dendritic cell vaccines and polyclonal T cell therapy as a novel immunotherapy against gliomas, as explored in a study by Flores et al. [174]. The goal of recruiting T cells towards the TME was successfully achieved, but researchers were not able to induce the differentiation of HSPCs into the final form of effector cells. This was due to IFN-gamma released by T cells once they reached the TME. Indeed, IFN-gamma is able to induce the differentiation of HSPCs into DCs, which, in turn, are very effective at presenting tumor antigens to the same T cells that released IFN-gamma in the TME [175]. This is a typical circular mechanism that works based on positive feedback. In another study on gliomas and glioblastomas, two cancers known for exhibiting a strong resistance to immunotherapy, PD-1 was blocked by means of monoclonal antibodies. After recruiting HSCs into the tumor site, the cell quota exhibiting the CCR2 receptor was found capable of both originating DCs and exerting a certain antitumor activity. In fact, surprisingly, mice receiving HSC infusions were also able to partially revert tumor resistance to cellular therapy [176].

The increasingly strong resistance to immune treatments exhibited by cancer is also due to the suppression of MDSC-mediated T cells’ activity, negatively impacting the effectiveness of immunotherapy and patient outcomes [177,178]. The presence of circulating MDSCs mainly correlates to the tumor stage, as described by recent research investigating the interplay between tumor stage, PD-1, and CTLA4. Indeed, blocking the MDSCs seems to strengthen PD-1 checkpoint inhibition, with a pivotal role played by CXCR2 receptors in this process [179].

Other studies focused on the role of MDSCs in patients treated with Ipilimumab, an antibody directed against the CTLA4 immune checkpoint. Meyer et al. [180] found that in patients treated for melanoma with Ipilimumab, the subpopulation composing the MDSCs’ lineage could be used as a predictive marker. Interestingly, subjects bearing a reduced amount of Lin− CD14+ HLA-DR− exhibited a better clinical response.

Tarhini et al. [181] studied a cohort of patients with advanced melanoma, undergoing neoadjuvant therapy with ipilimumab. Eventually, they could observe an inverse proportionality between the number of Treg cells in the TME and treatment effectiveness, highlighting the ability of this drug to modulate Treg cells. Furthermore, after one-year treatment, patients exhibiting progression-free survival showed an evident decrease in MDSC subpopulation Lin1-/HLA-DR-/CD33+/CD11b+.

In the case of hepatocellular carcinoma (HCC), TAMs are the predominant immune cell population in cancerous hepatic tissue and play a crucial role in determining tumor invasiveness, growth rate, immunosuppression, and angiogenesis. In particular, the TAM subpopulation of Kupffer cells (KCs) is responsible for the creation of an immune-tolerant environment since they express PD-L1 and are able to maintain low levels of costimulatory factors like CD80 and CD86 by priming Treg cells and releasing immunosuppressive cytokines such as IL-10 and TGFβ [182]. Moreover, in HCC, TAMs express high levels of SLC40A1, a marker gene encoding ferroportin, which activates Toll-like receptor (TLR) stimulation to produce IL-1β, IL-6, and IL-23, thus shifting innate immunity processes toward tumor-favoring activity [183]. Strategies targeting TAMs focus on blocking their recruitment, eliminating them, or altering their function. CSF-1 and its receptor (CSF1-R) pathway are critical for macrophage survival and differentiation. Clinical trials across various cancer types have explored drugs that inhibit this CSF-1/CSF1-R pathway, utilizing both monoclonal antibodies and small-molecule inhibitors [184]. Additionally, combining the CXCL12-CXCR4 inhibitor AMD3100 with Sorafenib and PD-1 blockade has demonstrated antitumor effects in a mouse HCC model [185,186]. Current research [187] is also evaluating potential survival benefits in HCC patients resulting from the combination of Nivolumab with either a CCR2/5 inhibitor or an anti-IL8 antibody (BMS-986253), administered either pre- or post-surgery.

Concerning neutrophils, the WHO histopathological classification assessed the neutrophil-rich variant of HCC as one of the most aggressive, although molecular and therapeutic correlates for this specific subtype of HCC remain poorly investigated [188]. 

In recent years, research on innate immunity has expanded the range of factors that contribute to cancer development, progression, and response to treatments. Dysfunctional and emergency myelopoiesis have garnered increasing attention. Specifically, in relation to the liver and HCC, several reasons support the investigation of dysfunctional myelopoiesis. First, the liver plays a central role in immune response due to its physiological functions. Second, HCC is typically an inflammatory cancer in the majority of cases, with numerous studies confirming that immune infiltrates significantly influence prognosis [189]. Third, the rise of HCC caused by metabolic syndrome, along with the established interplay between dysmetabolism, dysfunctional myelopoiesis, and cancer, compels us to focus on understanding these self-maintaining vicious cycles for prevention and treatment. Indeed, dysfunctional myelopoiesis in HCC development, progression, and treatment response remains under-researched, also due to the analytical challenges in isolating and preserving granulocytes. Notwithstanding, indirect evidence—such as the predictive role of the neutrophil-to-lymphocyte ratio (NLR) in HCC patients [190]—along with preclinical data support further investigation into this branch of the immune response to identify both biomarkers and therapeutic targets.

Exploring the presence and treatment-induced changes of dysfunctional myelopoiesis adds another layer of complexity to the understanding of the mechanisms driving responses to immune checkpoint inhibitors. However, in the case of PD1 blockade, preclinical data strongly support the pivotal role of PD1 inhibition in the myeloid compartment in modulating T cell responses [171]. Interestingly, the conditional knock-out of PD1 in the myeloid compartment was more effective in suppressing tumor growth than T cell-specific PD1 inhibition. Strauss et al. demonstrated not only that does cancer-driven emergency myelopoiesis express PD1 on myeloid precursors, but also that PD1 inhibition in these precursors increases the production of effector myeloid cells as well as the functionality of effector memory T cells, suggesting that myeloid precursors are key targets of antitumor immunity triggered by PD1 blockade. This study highlights the central role of the myeloid compartment in anticancer immunity and confirms PD1′s involvement in M1 polarization and the phagocytic function of TAMs. Remarkably, conditional PD1 knock-out in the myeloid compartment proved to be more effective than PD1 knock-out in lymphocytes. Seminal studies on PD1 blockade have demonstrated how it dictates the antitumor immune response [171], with the role of innate immune response surpassing that of adaptive immunity, which tends to remain the primary focus of research.

In the case of HCC, many questions remain unanswered regarding the mechanisms driving the response to immunotherapy, including the actual role of emergency and dysfunctional myelopoiesis in the modulation of immune-checkpoint inhibitor (ICI) effectiveness. Key concerns include metabolic dysfunctions in emergency myelopoiesis and aberrant activation of the wnt/beta-catenin pathway. Both factors are thought to inhibit the effects of ICIs [191,192] and are present in a significant proportion of HCC cases. Taken together, these findings underscore the importance of exploring emergency myelopoiesis in the context of HCC immunotherapy to better understand and target resistance mechanisms. Among recent studies on myelopoietic populations aimed at identifying early prognostic biomarkers to predict the outcome of cancer therapies, our research has shown that specific myeloid populations, such as granulocytes, act as useful early predicting markers for immunotherapy outcomes in HCC [193].

In patients with advanced HCC, we have observed several indicators of dysfunctional myelopoiesis, including an increased percentage of PD1+ and CTLA4+ peripheral granulocytes compared to matched cirrhotic patients (to be published). Remarkably, a higher percentage of baseline PD1+ granulocytes before starting treatment predicted a lack of response to immunotherapy [193]. These findings align with the pivotal role of the PD1 pathway in emergency myelopoiesis, as illustrated above in preclinical models [171]. Interestingly, in our HCC patients cohort, the percentage of PD1-positive granulocytes displayed better predictive performance in the setting of atezolizumab–bevacizumab treatment when compared to NLR [193].

Other research has found that the systemic immune-inflammation index (SII) depicts the situation of patients’ immune system. High levels of SII have been found to correlate with poor prognosis in HCC patients following resection [194]. Moreover, myeloid markers expressed on specific myeloid populations (MDSCs, granulocytes, dendritic cells, and macrophages) have been investigated to design a prognostic myeloid signature for HCC [195]. Further research is needed to investigate whether any differences exist between peripheral and TME subpopulations of myelopoietic biomarkers, to enhance the early design of patient-customized therapies.

**Figure 4:**
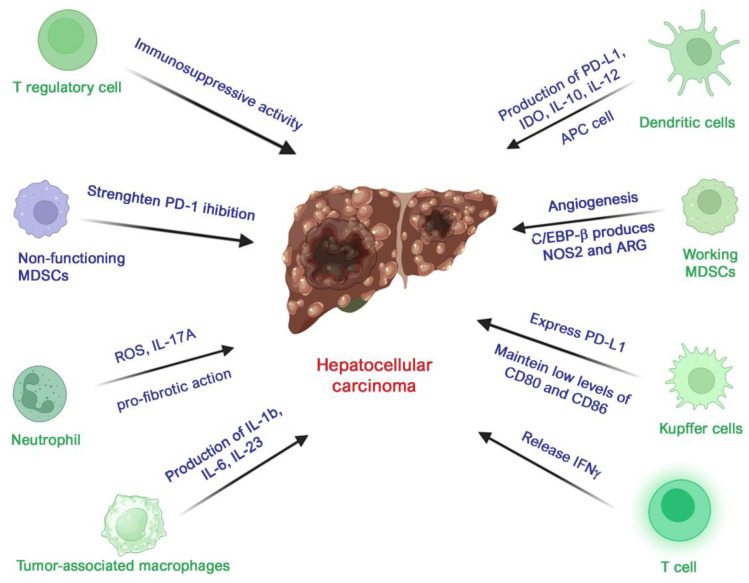
Schematic representation of major effects of main immune system cell lines on HCC. Effects are reported in terms of cytokine production and principal favoring/suppressive influence exerted by functioning (green) and non-functioning (purple) immune cell types on HCC. Created with Biorender.com and then edited.

As outlined above, MDSCs play a number of roles in the development of many cancers, including the enhancement of angiogenesis and promotion of pre-metastatic niches [196]. Moreover, they steer the activation of specific pathways, including autophagy, CREB signaling, cell cycle, CSF1, G-protein, GnRH, IFN-γ, IL-3 activation, MAPK, and mTOR [197]. However, how these cells act within the specific case of HCC’s tumor microenvironment has not yet been well established. Studies on MDSCs in patients with liver resection for HBV-related HCC found that tumor resection was followed by a decrease in the number of MDSCs and CD8+ T cells but had no effects on CD4+ T cells [198]. Conversely, high levels of MDSCs after resection correlated with early cancer recurrence. Recent studies have investigated the use of specific molecules like RORC1, PIR-B, ILTs, and C/EBP to modulate MDSCs. In HCC, MDSCs’ immunosuppressive activity was enhanced by the ability of C/EBP-β to regulate the expression of immunosuppressive genes like NOS2 and ARG [199]. Considering the many ways in which MDSCs are involved in cancer development and progression, they make a promising target for immunotherapy. However, the molecular mechanisms underlying MDSCs’ inhibiting action in HCC remain not fully understood. Moreover, considering the heterogeneity that characterizes MDSCs, further research should aim to investigate the outcomes of using different MDSC subpopulations as targets (Figure 4).

## 5. Conclusions

In conclusion, this review has described the crosstalk between emergency myelopoiesis and host metabolism. In the case of HCC, it has been observed that metabolic dysregulation might trigger the production of inflammatory mediators that enhance the production of cytotoxic myeloid NK (MyeNK) cells and their recruitment in the liver [200]. Considering the strict correlation between metabolic syndrome-associated non-alcoholic steatohepatitis (NASH) and the development of HCC, it is of utmost importance to investigate the interplay between myelopoiesis and metabolism in the HCC TME and HCC immune treatment.

## Data Availability

Not applicable.
